# Advance Monitoring of COVID-19 Incidence Based on Taxi Mobility: The Infection Ratio Measure

**DOI:** 10.3390/healthcare12050517

**Published:** 2024-02-21

**Authors:** Jesus S. Aguilar-Ruiz, Roberto Ruiz, Raúl Giráldez

**Affiliations:** School of Engineering, Pablo de Olavide University, 41013 Seville, Spain; robertoruiz@upo.es (R.R.); giraldez@upo.es (R.G.)

**Keywords:** COVID-19, virus spread, virus incidence monitoring, mobility, decision making

## Abstract

The COVID-19 pandemic has had a profound impact on various aspects of our lives, affecting personal, occupational, economic, and social spheres. Much has been learned since the early 2020s, which will be very useful when the next pandemic emerges. In general, mobility and virus spread are strongly related. However, most studies analyze the impact of COVID-19 on mobility, but not much research has focused on analyzing the impact of mobility on virus transmission, especially from the point of view of monitoring virus incidence, which is extremely important for making sound decisions to control any epidemiological threat to public health. As a result of a thorough analysis of COVID-19 and mobility data, this work introduces a novel measure, the Infection Ratio (IR), which is not sensitive to underestimation of positive cases and is very effective in monitoring the pandemic’s upward or downward evolution when it appears to be more stable, thus anticipating possible risk situations. For a bounded spatial context, we can infer that there is a significant threshold in the restriction of mobility that determines a change of trend in the number of infections that, if maintained for a minimum period, would notably increase the chances of keeping the spread of disease under control. Results show that IR is a reliable indicator of the intensity of infection, and an effective measure for early monitoring and decision making in smart cities.

## 1. Introduction

On 31 December 2019, the World Health Organization (WHO) China Country Office was informed of cases of pneumonia of unknown etiology detected in Wuhan City, China. The Chinese authorities identified a new type of coronavirus (novel coronavirus, nCoV), which was isolated on 7 January 2020. Later, the virus was named SARS-CoV-2 (severe acute respiratory syndrome coronavirus 2), causing the infamous COVID-19 (COrononaVIrus Disease 2019) disease. The WHO on 11 March 2020 declared the novel coronavirus outbreak a global pandemic and on 4 May 2023 stated that COVID-19 no longer constituted a public health emergency of international concern. After more than three years, a retrospective look leads us to an unsatisfactory judgment on the many diverse measures and policies adopted to combat the disease since its inception.

Undoubtedly, human to human interaction is the most prolific method of transmission of the virus. The only fully effective measure to eradicate the virus in the world is maximizing social distancing, i.e., global self–isolation. However, this measure entailed bringing the world to a complete halt for a period (e.g., three weeks). This decision requires an exercise of collective sanity that humanity is not capable of achieving.

Consequences of the first outbreak were underestimated by most, if not all, countries, partly due to the great lack of knowledge of the virus and to major concerns over economic impact. However, history has taught us that in similar situations, adopting a conservative attitude is preferable to a tolerant one. The question that should be asked now is whether the impact of a global categorical decision would have been less than what we have been stoically enduring since early 2020, having already experienced a death toll of about 7 million people (with many millions more cases likely not officially reported—in May 2023, the WHO stated that this figure could be seven times higher (WHO ‘Director-General’s opening remarks at the media briefing—5 May 2023’. https://www.who.int/director-general/speeches/detail/who-director-general-s-opening-remarks-at-the-media-briefing---5-may-2023 (accessed on 26 September 2023)). What is very clear is that if the isolation of everyone cannot be achieved, the next level is to reach domestic isolation, i.e., the confinement of family units. Should any member be allowed to violate this containment, though, the measure loses its strength. This is exactly what has happened and why hygiene, self-protection and social distancing rules became prominent.

The dynamic nature of cities has led to substantial changes in urban environments, making the analysis of geo-spatial information more complex [1]. The current capacity for data generation provides a unique opportunity to comprehend the dynamics of cities and the behavior of their inhabitants. This information holds invaluable knowledge for understanding behavioral patterns in the face of anomalous situations, such as those caused by the pandemic.

Urban mobility encompasses a wide range of transportation options and sources that help people move around cities efficiently. Some studies related to COVID-19 have focused on public transportation, such as urban rail systems [2], public buses [3], a combination of them (e.g., public transit, ride-shares, bikes, and walking [4]), or gendered mobility patterns [5]. In principle, knowing more about the relationship between increased or decreased mobility and the incidence of COVID-19 will help us to act more effectively in future pandemics. Nevertheless, interest in many works has focused on the impact of COVID-19 on mobility, and few efforts have been directed at assessing how regulating mobility rates can have positive effects on controlling virus transmission. Examples of these studies have focused on Taiwan [6], Poland [7], the United States [8], India [9], Spain [10,11], Japan [12], the United Arab Emirates [13], Saudi Arabia [14], Greece [15], China [16,17], Indonesia [18], Austria [19], Italy [20], Portugal [21,22], South Africa [23], Costa Rica [24], and Australia [25], among others.

The mobility of individuals during the COVID-19 pandemic differs substantially from country to country [26,27]. Numerous studies have examined the relationship between human mobility and COVID-19 transmission using spatio-temporal data. Empirical evidence suggests that lower rates of COVID-19 infection and mortality are associated with increased levels of social distancing and reduced usage of public transit modes [28]. Implementing restrictions on human mobility has been effective in reducing COVID-19 transmission, although the effectiveness of such policies can vary temporally and spatially across different stages of the pandemic [29]. For instance, a study based on zip code data from five U.S. cities estimated that the total cases per capita decreased by 19% for every 10% decrease in mobility [30]. In a study covering 34 OECD countries, Singapore, and Taiwan, it was found that reductions of up to 40% in commuting mobility were associated with decreased cases, particularly during the early stages of the pandemic, in two-thirds of the countries [31]. Another study analyzing data from 52 countries also highlighted a strong link between mobility measures and transmissibility, supporting the benefits of population-wide social distancing interventions [32]. In the United States, a reduction of 10% in mobility was associated with a substantial decrease in case growth (17.5%) two weeks later [33]. In Europe and the U.S., the impact of social distancing measures resulted in a reduction of 20–40% in infection rates for most European countries and 30–70% for the U.S., observed two to five weeks after the implementation of mobility reduction [34]. Additionally, mobile phone location data were used in a longitudinal study to analyze population mobility and COVID-19 incidence across 314 Latin American cities. The findings indicated that a 10% lower weekly mobility was associated with an 8.6% lower incidence of COVID-19 in the following week [35]. Analysis of mobility data in England and Wales revealed a strong correlation between mobility reductions and lower excess mortality, considering a five-week lag between the two variables [36]. Similarly, a study of the most affected counties in the U.S. detected decreases in COVID-19 case growth rates due to changes in mobility patterns, which dropped by 35–63% relative to normal conditions. However, the impact was not immediately perceived, and reductions in case growth rates were observed up to 9–12 days later [37]. Other research has identified positive associations between mobility growth patterns and the increase in the number of COVID-19 cases, with lags of 5 to 7 weeks during the fast growth phase of the pandemic [38]. Interestingly, the number of new cases daily has been linked to trips taken more than two weeks before, suggesting that commonly set 14-day mobility restrictions for quarantine may potentially underestimate the effectiveness of containment policies [39].

Social distancing is effective in reducing virus spread. Therefore, maximizing social distancing in transportation means minimizing transmission risks. In this context, private vehicles gained significance among people in need of transportation. However, in big cities where parking places are scarce or expensive, private cars are not as convenient, and taxis become an excellent representation of global mobility. Several papers have been published with the aim of examining the correlation between taxi mobility and the impact of COVID-19 in specific contexts. For instance, in NYC, researchers focused on identifying exhaust emission patterns and changes in urban mobility during the lockdown period in 2020 [40]; another study explored the profound impact of the COVID-19 outbreak on travel patterns [41]. In Chicago, research has delved into the impact of the pandemic on the structure of the mobility network of taxis [42], and another study investigated the variation in taxi tipping rates according to COVID-19 intensity [43].

This study focuses on mobility behavior and on how urban mobility is related to virus prevalence, both in terms of infections and deaths. Hygiene and self–protection measures depend on individuals’ willingness, the availability of resources in the market, and the economic capacity to obtain necessary materials like face masks and hydroalcoholic gels. Undeniably, maximum social distancing (zero mobility) for a short period of time would probably have annihilated the virus, unless the virus could survive for a long time under certain external conditions or could reproduce on its own without a host.

To analyze the behavior of individuals as subjects in motion who are capable of contacting and potentially infecting others, New York City (NYC) has been selected as a representative urban area that mirrors the dynamics of mobility in many metropolises worldwide. There are various sources of information that could be provided to an analytical model to comprehend mobility dynamics during a pandemic, ranging from individual-level data generated at any time (representing a vast volume of information) to statistically significant samples obtained as subsets. In a modern, technologically advanced city, transportation serves as a highly reliable indicator of mobility patterns. Data generated by cab trips are valuable as they encourage distancing compared to other means of public transportation, such as buses or subways. To provide a bounded temporal context for the analysis of the pandemic’s irruption and its relationship to mobility, data generated by NYC cabs during the year 2020 (the period of greatest uncertainty in which the first wave occurred) will be analyzed. This analysis could be easily extrapolated to other years, as well as to other metropolises. Moreover, its reproducibility in the event of a new pandemic outbreak is significant.

As mentioned above, most studies have focused on the impact of COVID-19 on mobility, but not much research has focused on analyzing the impact of mobility on virus transmission, especially from the point of view of monitoring virus incidence, which is extremely important for making sound decisions to control any epidemiological threat to public health. Therefore, the goal of this research is to show that it is also possible to measure the impact of mobility policies on COVID-19 incidence in advance, which is very useful for controlling the spread of the disease.

The rest of the document is organized as follows: Section 2 presents the sources of information (COVID-19 and mobility data) and introduces the Infection Ratio measure; Section 3 shows the results of the analysis of mobility data from New York City cabs during 2020 and the evolution of COVID-19 data in the same city; Section 4 discusses the relationship between infections and mobility, highlights the strong association between both variables, and introduces the role of the Infection Ratio in monitoring the impact of the pandemic; finally, the main conclusions and future work are described in the last section.

## 2. Methods

### 2.1. COVID-19 Data

The first case of the COVID-19 pandemic in NYC was confirmed on 29 February 2020 (as the first laboratory-confirmed case), though later research showed that the novel coronavirus had been circulating since January [44]. The Health Department provides a repository that contains updated data on coronavirus disease 2019 (COVID-19) in NYC. The numbers of infected and deceased people were smoothed by applying seasonal trend decomposition via locally estimated scatterplot smoothing (STL) [45] to the original data in order to isolate trends from seasonal and noise effects.

### 2.2. Mobility Data

NYC boasts one of the largest taxi mobility databases, with easy full access from the New York City Government web site [46]. The data used were collected and provided to the NYC Taxi and Limousine Commission (TLC) by technology providers authorized under the Taxicab and Livery Passenger Enhancement Programs (TPEP/LPEP). Taxi trip records include fields capturing pick-up and drop-off dates/times, pick-up and drop-off locations, trip distances, itemized fares, rate types, payment types, and driver-reported passenger counts.

**Figure 1:**
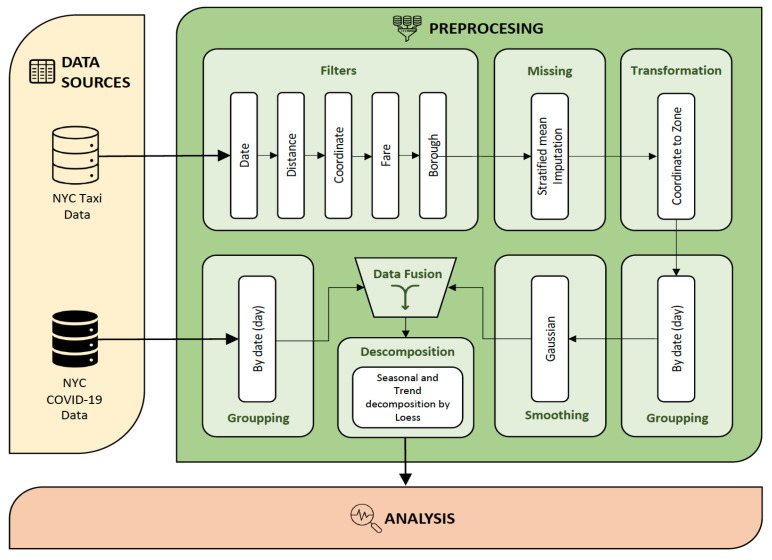
Diagram of the methodology applied in data preprocessing.

**Figure 2:**
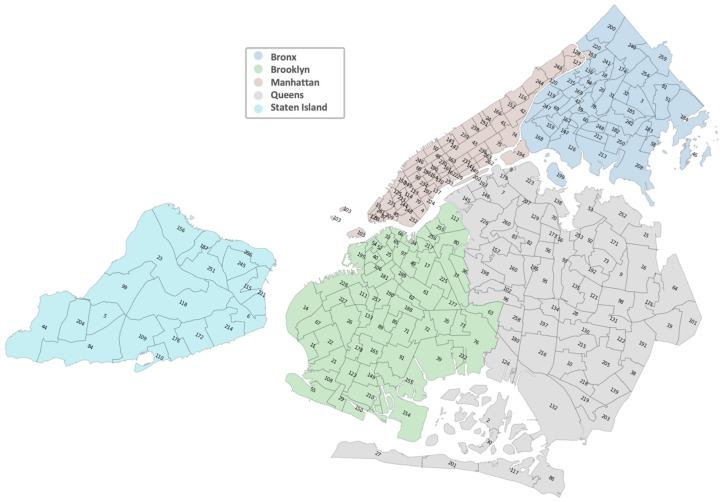
Zones in Manhattan, the Bronx, Brooklyn, Queens, and Staten Island.

The data included trips from the five boroughs of NYC (Manhattan, the Bronx, Brooklyn, Queens, and Staten Island) and amounted to 28,667,765 trips. Extensive cleaning was necessary to ensure a high-quality dataset for analysis, as outlined in Figure 1 (modules named Filters, Missing and Transformation). The Taxi Zone Shape file was used to filter pick-up and drop-off locations identified by latitude and longitude to exclude trips outside the area of interest. Trips with distances smaller than 0.1 or greater than 85 miles (the longest one-way trip within the area is approximately half this distance) were removed as they were considered far outliers. Trips with fares smaller than USD 1 were also eliminated, and all fare inconsistencies were filtered based on the day of the week and time. Subsequently, all coordinates were transformed into zone codes, as depicted in Figure 2, with colors corresponding to the respective borough. The final dataset included trips that had both pick-up and drop-off within the zones identified in the map, amounting to a total of 28,102,750 trips (a decrease of about 2%).

### 2.3. The Infection Ratio

Basic (R0) or effective (Rt) reproduction numbers are epidemiological metrics used to measure the contagiousness or transmissibility of infectious agents. However, the values are often estimated by using ordinary differential equations. Therefore, the calculation and interpretation are not straightforward, and although they remain valuable concepts, they should be applied with caution [47] and considering a long list of recommendations that depend on the phase of the pandemic [48]. In fact, many countries have adopted concepts that make it easier for society to understand the status and evolution of the pandemic. For example, the cumulative incidence rate per 100,000 population has been postulated as an understandable indicator of the improvement or worsening of the pandemic situation.

Taking into consideration that the latent period is about 3.1 days (immediately after infection), the prodromal infective period is about 2 days, and the early infective period lasts around 5 days [49], it becomes possible to visualize the evolution of the pandemic by analyzing a novel measure introduced in this work: the Infection Ratio (IR). The IR is calculated as the ratio of people infected during a period of three days and not capable of infecting others (denoted λ) to the number of people infected during the preceding 14 days with the potential to infect others (denoted µ).

(1) $$Infection\;Ratio\;(IR)=\frac{\lambda }{µ}$$

The advantage of the IR lies in its ability to account for potential biases in the number of infections, particularly underestimation. The assumption is that this bias remains constant over the three days when infected persons are recorded and the previous fourteen days when individuals with the capacity to infect are counted, i.e., it is not likely that reported cases have varying levels of underestimation over the 17-day period. 

Let π be the infection underestimation factor. Typically, both λ and µ, representing reported infection values, tend to be lower than the actual values. Should there be any variation in underestimation over time, it is expected to be gradual, given the consistent nature of reported values during this duration. Including π in Equation (1): IR = (λ + πλ)/(µ + πµ) = λ/µ implies that the true values are assumed to be π percent higher. Therefore, such variations are not substantially impacting the calculated IR, and as a result, the bias has a negligible effect on the IR values.

## 3. Results

**Figure 3:**
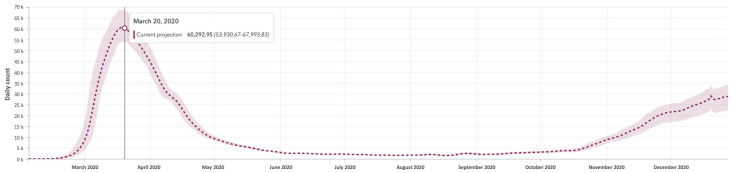
Estimated number of infections during 2020 for New York State following the IHME model (source accessed on 15 November 2021: https://COVID19.healthdata.org/).

A critical limitation in our understanding of the COVID-19 pandemic is that we cannot trust statistical data provided by local and state governments, since the true number of infections does not correspond to the number of confirmed tests (many infected people were never tested because they did not have access to testing or had no symptoms and did not even know they were infected, though they were still able to transmit the virus). Such asymptomatic infections were estimated to be 15–70% of total infections (source: Centers for Disease Control and Prevention, U.S. Department of Health and Human Services [50]), although more precise models for NYC state that the proportion of symptomatic cases ranges from 13 to 18% [51], i.e., 82–87% of cases are asymptomatic. Several models have been developed to estimate the true daily number of infections, and the figures are surprisingly generous. For example, Figure 3 shows estimates from the Institute for Health Metrics and Evaluation (IHME) model [52] for the year 2020 for New York State. Estimations are sometimes about four times greater than the reported data. In addition, the reported case figures on a given date do not necessarily show the number of new cases on that day, due to delays in updating data, and not all countries use the same criteria for counting infections and deaths. For example, by the end of December 2020 in Germany, a country with around 84 million inhabitants, 1.7 million positive cases and some 33,000 deaths had been reported, while in Bangladesh, a country with 165 million inhabitants, only half a million infected people had been informed (data gathered and processed from the COVID-19 Dashboard by the Center for Systems Science and Engineering at Johns Hopkins University) [53].

In general, COVID-19 data have been collected worldwide on such diverse criteria that they cannot provide general, reliable conclusions based on quantitative measures; instead, qualitative conclusions should be drawn with higher degree of abstraction with respect to the data in specific regions, i.e., based on the behavior of the data (relative measures) rather than on their values.

**Figure 4:**
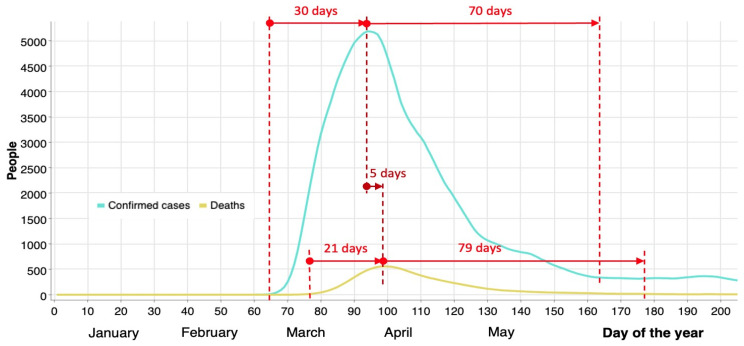
Confirmed cases and deaths during the first wave (2020) in NYC.

Figure 4 shows the evolution of COVID-19 during the first wave in 2020 in NYC. It shows the approximate number of days from the beginning (zero values) to the maximum peak value (30 and 21 days, for infected and deceased persons, respectively), and then from the maximum to a stable minimum value (70 and 79 days, respectively). The total number of confirmed cases during the first 100 days (30 + 70) is 209,000, while the number of deaths (21 + 79) is 18,400 (almost 9%).

From the perspective of decision making timeliness, it does not appear reasonable that it takes about 3 weeks to implement measures to mitigate the number of infections, and an additional 16 days to observe their impact. This delay in response does not demonstrate alacrity and decisiveness on the part of political authorities. By reducing this timeframe from three weeks to one week, the number of infections during the first wave could have been drastically reduced. An analysis involving 10 different countries found that fast decisions are critical, and if taken early enough, an up to 40% reduction in mobility would be sufficient to control the level of infections [54].

At this juncture, it is important to highlight that the probability density function of the incubation period, defined as the period (in days) from virus exposure to the onset of symptoms, does not follow a normal distribution; instead, it has been modeled using lognormal, Weibull, and Gamma distributions. As the incubation period is estimated to be between 4.5 and 5.8 days, and the onset of symptoms occurs between 9.7 and 14.2 days [55], to minimize the risk of infection requires a minimum isolation period of 14 days (95% CIs) from the time of infection (uncertainty increases towards the tail of the lognormal distribution).

**Figure 5:**
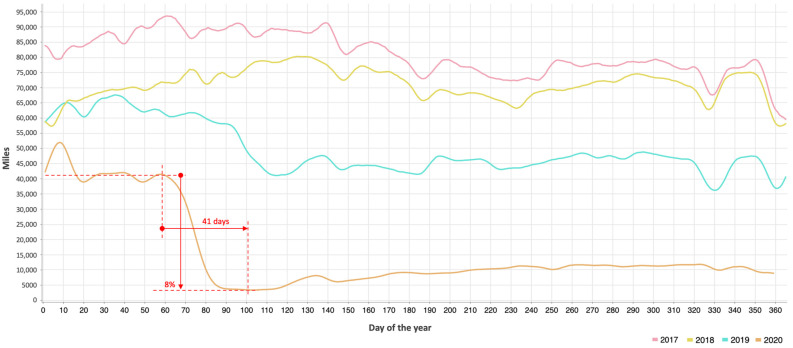
Daily distance traveled by cabs during 2017, 2018, 2019 and 2020 in NYC.

Figure 5 shows the curves of the daily distances traveled by cabs during the period from 2017 to 2020, inclusive. Gaussian smoothing using a 7-day sliding window with a central mean was applied to generate the curves, thus avoiding bias due to variations in mobility on weekdays and weekends. At first sight, the most notable aspect is the significant decrease in mobility that occurred progressively in 2020, lasting for 41 days and reaching a minimum equivalent to 8% of mobility with respect to the pre-pandemic period (baseline). This minimum was sustained for only 20 days, after which mobility slowly started to rise, ranging between 15% and 30% of the baseline throughout the second half of the year. 

**Figure 6:**
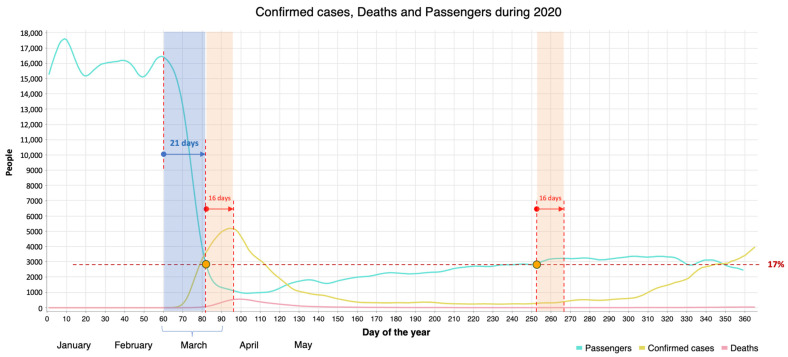
Confirmed cases, deaths and passengers during 2020 in NYC.

Analysis of the variability of mobility of individuals in relative terms can shed light on the impact of contact limitation on the number of infections. Instead of distances, Figure 6 depicts the number of taxi passengers versus the evolution of the number of infections and deaths. By identifying an approximate inflection point in the graph that denotes a deceleration in the growth rate of confirmed cases, an estimate is placed around day 82, aligning with mobility levels around 17% of normal (pre-pandemic) values. This specific point (marked in orange) signifies the onset of the declining phase leading up to the peak in confirmed cases, occurring 16 days later. Subsequently, mobility remains under 17% until day 252 (second point highlighted in orange). After this period, mobility surpasses 17%, coinciding with a resurgence in confirmed cases and stabilizing approximately 16 days later, followed by a significant increase.

## 4. Discussion

The adopted measures did not have an immediate impact, meaning that their implementation was not visibly reflected in population movement over the following days. For instance, when the state of emergency was declared on 7 March 2020, in New York State, mobility started to slowly decline, reducing to 10–20% towards the end of March [56], about a week before reaching the peak of infections. 

Several observations surface from Figure 6: (a) the 21 days prior to the initial orange point are highly dependent on the promptness of intervention measures, suggesting a possible need for rapid actions, which would have had a large impact on the area under the curve (i.e., the total number of infections); (b) understanding the impact of reduced mobility in NYC on the growth rate of confirmed cases (the first orange dot) aids in approximating the levels of sustained mobility necessary to prevent a recurrence similar to the subsequent surge in infections (the second orange dot). In order to significantly curtail the infection count, mobility needed to be restricted to a maximum of 17%. Conversely, surpassing this threshold resulted in a noticeable upswing in infections. Clearly, any value ranging between 8% (the minimum value reached in Figure 5) and 17% (indicating a shift in the trend in Figure 6) could be considered a viable critical threshold for mobility. The specific value of this threshold will depend on the region being studied but serves as a reference to illustrate the critical importance of mobility in determining a downward or upward trend change in the number of infections. Nonetheless, regardless of the threshold, it takes about two weeks for the consequences of reduced or increased mobility to begin to be felt.

**Figure 7:**
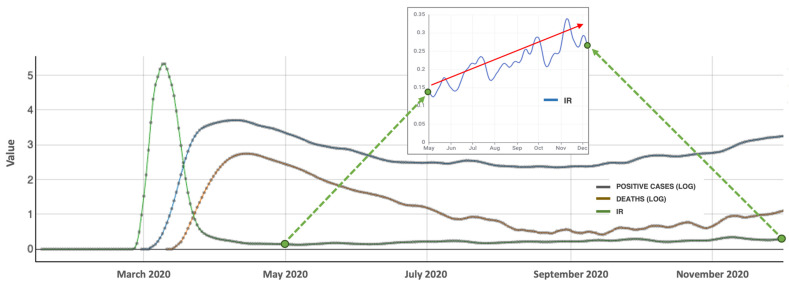
Logs of infected people (blue) and deaths (brown), and the Infection Ratio (green) during 2020. Small graph shows the increasing trend (red arrow) of the Infection Ratio (0.13–0.34) from May to December.

Plotting the IR for the period of interest yields a curve that serves as a reliable indicator of the evolution of the overall intensity of infection. In Figure 7, the IR clearly shows the tragic month of March. When the IR is greater than 1, it indicates that the power of infection during the next two weeks will surpass the current level. On 23 March (day 82), the IR falls below 1, leading to a decline in the number of infections two weeks later. From the beginning of May, the curve appears to stabilize, but a closer examination reveals oscillations with an increasing trend (see red arrow). By 8 September (day 252), the IR exceeds 0.25 again, meaning that for every four people infected during a two-week period, one person will be infected within the following three days. As observed in the figure, this increase in IR corresponds to a rise in the number of infected people and deaths. On the other hand, this situation is also reflected from day 252 onwards in Figure 6, when mobility exceeds 17%. Furthermore, the peak of IR (9–10 March) occurs approximately three weeks before the peak of positive cases (first week of April), indicating its importance as a measure for early decision making. Throughout different phases (waves) of the virus spread, close monitoring of indicators is essential, as their significance lies in the analysis of their evolution rather than the specific numerical values. A monitoring of the evolution of the IR from May to September would have revealed in advance the worsening of the situation.

As a result of this analysis, we can conclude that the use of direct measures such as the number of infections or deaths is not sufficiently effective for decision making, mainly due to the difference between reported and actual infections, as criteria for counting deaths due to COVID-19 have varied between countries (e.g., in Spain, deaths in nursing homes were not reported. (‘Poor data obscures COVID-19 death toll at Spain’s nursing homes’. REUTERS. 7 July 2020). However, the analyzed data clearly demonstrate an association between the degree of mobility restriction and the spread of the virus. The use of relative measures, such as mobility variability or the novel infection ratio, could be more effective in measuring the pulse of a pandemic, especially regarding the impact of social distancing on the number of infections. 

## 5. Conclusions

The mobility data are highly representative and of excellent quality, but the same cannot be claimed for the data on infections and deaths. Consequently, the data should be analyzed very carefully. Relying solely on the number of reported infections for decision-making is ineffective, as there could be a significant disparity between the reported and actual number of infections.

Mobility has proven to be an excellent indicator for monitoring the dynamics of a pandemic. Existing research has demonstrated a strong correlation between mobility patterns and fluctuations in the number of infections and deaths. However, less attention has been devoted to the impact of mobility on virus transmission and how controlling mobility may be a powerful tool for preventing the spread of the disease in advance.

Utilizing relative measures such as the Infection Ratio is valuable as it mitigates the impact of underreported cases and aids in effectively monitoring the virus’s spread while assessing the efficacy of implemented policies. Results show that the IR is a reliable indicator of the intensity of infection and an effective measure for early monitoring and decision making. Although this study is specific to the characteristics of the analyzed city, variations in values across different environments do not undermine the interpretation and quality of the obtained results.

Future research endeavors will focus on expanding the application of the Infection Ratio measure to diverse viral infections, populations of varying sizes, and geographical settings, provided that adequate and comprehensive datasets become available. These efforts aim to enhance the versatility and reliability of the measure for proactive monitoring and response strategies in potential future pandemics and infectious disease scenarios.

## Data Availability

Data are publicly available at Virus Disease: NYC Coronavirus Disease 2019 (COVID-19) Data (https://www1.nyc.gov/site/doh/covid/covid-19-data.page [accessed on 15 November 2021]); Mobility: N. Taxi and Limousine Commission (TLC) Trip Record Data, NYC (https://www1.nyc.gov/site/tlc/about/tlc-trip-record-data.page; [accessed on 15 November 2021]).
